# *Persicaria minor* (Huds.) Opiz Exhibits Antihypertensive Effects by Inhibiting the Angiotensin-Converting Enzyme/Angiotensin II Type 1 Receptor Pathway in Human Endothelial Cells

**DOI:** 10.3390/life14111486

**Published:** 2024-11-14

**Authors:** Nur Syakirah Othman, Nur Syahidah Nor Hisam, Amanina Athirah Mad Azli, Nur Izzati Mansor, Adila A. Hamid, Amilia Aminuddin, Nur Najmi Mohamad Anuar, Mohd Faizal Ahmad, Azizah Ugusman

**Affiliations:** 1 Department of Physiology, Faculty of Medicine, Universiti Kebangsaan Malaysia, Kuala Lumpur 56000, Malaysia; p118667@siswa.ukm.edu.my (N.S.O.); p104164@siswa.ukm.edu.my (N.S.N.H.); p132301@siswa.ukm.edu.my (A.A.M.A.); adilahamid@ppukm.ukm.edu.my (A.A.H.); amilia@ppukm.ukm.edu.my (A.A.); 2 Programme of Biomedical Science, Centre for Toxicology & Health Risk Studies, Faculty of Health Sciences, Universiti Kebangsaan Malaysia, Kuala Lumpur 50300, Malaysia; nurnajmi@ukm.edu.my; 3 Department of Nursing, Faculty of Medicine, Universiti Kebangsaan Malaysia, Kuala Lumpur 56000, Malaysia; nurizzatimansor@ukm.edu.my; 4 Cardiovascular and Pulmonary Research Group (CardioResp), Universiti Kebangsaan Malaysia, Bangi 43600, Malaysia; 5 Department of Obstetrics and Gynaecology, Faculty of Medicine, Universiti Kebangsaan Malaysia, Kuala Lumpur 56000, Malaysia; drmohdfaizal@ukm.edu.my

**Keywords:** angiotensin-converting enzyme, human umbilical vein endothelial cells, hypertension, phorbol 12-myristate-13-acetate, *Persicaria minor*

## Abstract

Overactivation of the angiotensin-converting enzyme (ACE)/angiotensin II type 1 receptor (AT1R) pathway leads to vasoconstriction and elevated blood pressure. *Persicaria minor* (Huds.) Opiz is an herbal plant known for its antioxidant, anti-hyperlipidemic, and anti-atherosclerotic properties, with bioactive compounds that exhibit antihypertensive effects. Therefore, this study aimed to evaluate the antihypertensive effects of the standardized aqueous extract of *P. minor* leaf (AEPM) through the ACE/AT1R pathway in human umbilical vein endothelial cells (HUVECs) induced with phorbol 12-myristate 13-acetate (PMA). HUVECs were stimulated with PMA to induce ACE, with or without AEPM or captopril treatment, for 24 h. Subsequently, ACE mRNA expression, ACE protein levels, ACE activity, angiotensin II levels, and AT1R expression were measured. The results demonstrated that AEPM treatment significantly reduced ACE mRNA expression, ACE protein levels, ACE activity, angiotensin II levels, and AT1R expression in PMA-induced HUVECs. The modulatory effects of AEPM on the ACE/AT1R pathway were comparable to those of captopril. Ex vivo experiments further confirmed that AEPM reduced the contraction responses of rat aortic rings to PMA. In conclusion, *P. minor* effectively inhibits the ACE/AT1R pathway in PMA-induced HUVECs, suggesting its potential as a natural antihypertensive agent.

## 1. Introduction

Hypertension, often referred to as the ‘silent killer’, remains a major global health concern, contributing to over 10 million deaths annually [1]. If left uncontrolled or untreated, it significantly increases the risk of cardiovascular diseases, leading to conditions such as stroke, myocardial infarction, and renal failure. One of the proposed mechanisms in the pathogenesis of primary hypertension is the disruption of the renin–angiotensin system (RAS), a hormonal system integral to the regulation of blood pressure and fluid homeostasis.

Studies have shown that components of the RAS are present in various tissues and cells, including blood vessels, the heart, liver, skeletal muscle, adipose tissue, neurons, lungs, and reproductive organs [2]. This localized form of the RAS, referred to as the tissue or local RAS, operates independently of the systemic RAS, exerting autocrine and paracrine effects within these tissues [2]. Local RAS activation plays a critical role in regulating physiological functions such as vascular tone and oxidative metabolism without relying on the stimulation of the systemic RAS. However, the local RAS may still interact with the systemic RAS to produce broader endocrine effects [3]. Notably, studies have also identified RAS components in endothelial cells, which are essential in controlling blood vessel tone and managing oxidative stress [4], further highlighting the significance of the local RAS in vascular health.

The RAS consists of several key components, including renin, angiotensinogen, angiotensin-converting enzyme (ACE), angiotensin I (Ang I), and angiotensin II (Ang II). Briefly, renin cleaves angiotensinogen into Ang I, which is subsequently converted into the major bioactive peptide, Ang II, through the action of ACE. ACE is widely distributed in endothelial cells lining blood vessel walls [5]. Excessive ACE expression and activity have been linked to the pathogenesis of hypertension [6]. Ang II binds to the Ang II type 1 receptor (AT1R), leading to vasoconstriction, increased blood pressure, and the promotion of inflammation and oxidative stress [7,8,9]. Inhibiting ACE reduces the conversion of Ang I to Ang II, which in turn decreases vasoconstriction and lowers blood pressure.

In vitro ACE inhibitory activity has emerged as an effective tool for screening the antihypertensive potential of substances or natural products [10]. In contrast, phorbol 12-myristate 13-acetate (PMA), a polyfunctional diterpene phorbol ester isolated from *Croton tiglium*, is commonly used to induce ACE activity in experimental models, both ex vivo and in vitro [11]. PMA acts as a protein kinase C (PKC) activator, mimicking the function of diacylglycerol (DAG), an endogenous activator of PKC. PKC activation promotes ACE gene transcription and secretion in endothelial cells, playing a significant role in the development of hypertension [12].

ACE inhibition is widely recognized as a critical strategy in managing hypertension [13]. ACE inhibitors, including captopril, lisinoporil, perindopril, and enalapril, are commonly prescribed for patients with hypertension [13]. However, these synthetic medications are associated with side effects such as kidney failure, angioedema, cough, and fetal developmental defects [11,13]. Additionally, certain synthetic ACE inhibitors, such as enalapril and perindopril, may undergo intra-cyclization, resulting in the formation of diketopiperazine derivatives. These derivatives can exist in various polymorphic forms, which may influence the drug’s stability, bioavailability, and overall performance [14]. Therefore, research on natural ACE inhibitors is essential to mitigate these side effects and holds potential as both a preventive measure and complementary therapy for hypertension. Numerous studies have shown that several flavonoids, such as quercetin, luteolin, and kaempferol, inhibit ACE activity [15]. Various herbs, including *Allium sativum*, *Cinnamomum verum*, and *Zingiber officinale*, have demonstrated ACE-inhibitory effects [16].

*Persicaria minor* (Huds.) Opiz (synonym: *Polygonum minus*) is an herbaceous plant native to Southeast Asia. It is commonly used as a food additive and flavoring agent. Extracts of *P. minor* have been shown to exhibit antioxidant [17], anti-atherosclerotic [18], and anti-hyperlipidemic properties [19]. *P. minor* demonstrates other pharmacological effects, including immunomodulatory, anti-proliferative, and anti-inflammatory activities [20,21]. Toxicity studies have confirmed that *P. minor* is safe for consumption. Oral administration of an aqueous extract of *P. minor* leaves to Wistar rats for 28 days showed a no-observed-adverse-effect level (NOAEL) greater than 1000 mg/kg of body weight [22].

*P. minor* contains various active compounds, including quercitrin, quercetin, hyperoside, astragalin, apigetrin, isoquercetin, gallic acid, and miquelianin [21]. Previous studies have demonstrated that quercetin, myricetin, and gallic acid possess ACE inhibitory effects and antihypertensive activity [23,24,25]. However, the effect of *P. minor* on the ACE/AT1R pathway remains unexplored. Therefore, this study aimed to investigate the effects of *P. minor* on the ACE/AT1R pathway in PMA-induced endothelial cells and to validate its vasorelaxant effect ex vivo. The results could enhance our understanding of the potential of *P. minor* as an ACE inhibitor, which may subsequently be developed as a preventive measure and complementary treatment for hypertension.

## 2. Materials and Methods

### 2.1. Standardized Aqueous Extract of P. minor Leaf (AEPM) Preparation

*P. minor* leaves were authenticated by a plant taxonomist from the Institute of Bioscience, Universiti Putra Malaysia (specimen voucher number: MFI 0112/19). The AEPM used in this study was provided by Biotropics Malaysia Berhad. The extract was standardized using high-performance liquid chromatography (HPLC) to contain at least 0.59% quercetin-3-*O*-glucuronide and 0.27% quercitrin, according to Biotropics Malaysia Berhad’s in-house HPLC protocol [26]. In brief, fresh *P. minor* leaves were dried in an oven to achieve a moisture content of less than 10%, after which they were cut into small pieces. Extraction was performed by soaking the leaves in water at a ratio of 1:20 (*w*/*v*), followed by two cycles of percolation at 80 °C for 4 h. The extract was subsequently filtered, evaporated, and freeze-dried until the water content was less than 8%. The HPLC fingerprint of AEPM was acquired using a Kinetex 1.7 µm column (C18, 2.1 × 150 mm). The mobile phase consisted of solvent X (0.10% formic acid in water) and solvent Y (0.10% formic acid in acetonitrile), mixed according to a linear gradient ranging from 5 to 89% solvent X and from 95 to 11% solvent Y [26]. Peaks corresponding to quercetin-3-*O*-glucuronide and quercitrin, with retention times (R*_t_*) of 7.332 and 14.632 min, respectively, were confirmed by comparing their R*_t_* and UV spectrum with reference standards (Supplementary Figure S1).

### 2.2. HUVEC Isolation and Culture

This study received approval from the Ethical Research Committee of Universiti Kebangsaan Malaysia (approval number: UKM PPI/111/8/JEP-2019-671, dated 25 October 2019). Human umbilical cords were obtained with informed consent from healthy individuals in the labor ward at Hospital Universiti Kebangsaan Malaysia. Human umbilical vein endothelial cells (HUVECs) were isolated using the collagenase perfusion method, as previously described [11]. In brief, the isolation process involved treating the umbilical cords with 0.1% collagenase (Worthington Biochemical Corporation, Lakewood, NJ, USA) and culturing the cells in endothelial cell medium (ScienCell Research Laboratories, San Diego, CA, USA) at 37 °C in a humidified atmosphere of 5% CO_2_ and 95% air. All experiments were performed using HUVECs in passage 3, once they reached 80% confluency.

### 2.3. In Vitro Study Protocol

HUVECs in passage 3 were seeded into 6-well plates at a density of 1 × 10^5^ cells per well. After 48 h, when the cells reached approximately 80% confluency, they were divided into seven groups: an untreated control group, a group treated with 400 µg/mL AEPM alone, a group treated with 200 nM PMA to induce ACE [11], and groups receiving 200 nM PMA combined with 200, 300, or 400 µg/mL AEPM. Additionally, a group treated with 200 nM PMA and 0.06 µM captopril [11] served as the positive control. All treatments were administered concurrently for 24 h. After the 24 h treatment period, HUVEC samples were collected for further analysis.

### 2.4. Determination of ACE and AT1R mRNA Expression in HUVECs Using Quantitative Real-Time Polymerase Chain Reaction (qPCR)

The mRNA expression of ACE and AT1R were quantified using qPCR, following a previously established method [27]. Total RNA from HUVECs was isolated using TRI Reagent (Molecular Research Centre, Cincinnati, OH, USA) according to the manufacturer’s protocol. The purity and concentration of the extracted RNA were assessed using a spectrophotometer. Complementary DNA (cDNA) was synthesized using a QuantiNova™ reverse transcription kit (Qiagen, Hilden, Germany), adhering to the provided instructions. The qPCR primers were designed using Primer 3 software (http://frodo:wi.mit.edu/cgi-bin/primer3/primer3-www.cgi, accessed on 15 November 2020) based on sequences available in the NCBI GenBank database (Table 1). Glyceraldehyde-3-phosphate dehydrogenase (GAPDH) served as the internal control for normalization. The qPCR was carried out on a Bio-Rad CFX96 cycler (Bio-Rad Laboratories, Hercules, CA, USA), with an initial denaturation at 95 °C for 3 min, followed by 40 cycles at 61 °C for 30 s, 95 °C for 1 min, 55 °C for 1 min, and 70 cycles at 60 °C for 10 s, concluding with a cooling step at 4 °C. The relative expression of ACE and AT1R was determined using the 2^−∆∆CT^ method [11]. 

### 2.5. Measurement of the ACE and Ang II Protein Levels in HUVECs Using Enzyme-Linked Immunosorbent Assay (ELISA)

The levels of ACE and Ang II proteins were measured using Human ACE and Ang II ELISA kits (FineTest, Wuhan, China) following the manufacturer’s guidelines. HUVEC lysates were used for the measurement of ACE protein, while the culture supernatants were used for Ang II protein analysis. Samples and standards were added in duplicate to 96-well plates pre-coated with specific antibodies. After a 90 min incubation, a biotin-labeled detection antibody was introduced. The plates were washed three times before adding a streptavidin–horseradish peroxidase conjugate solution. Following this, the 3,3′,5,5′-tetramethylbenzidine substrate was added to the wells. After 30 min, the reaction was halted by adding a stop solution. The optical density of each well was read at 450 nm using a microplate reader. Standard calibration curves for ACE and Ang II were generated from the optical density readings, and the protein concentrations in the HUVEC samples were determined based on these curves.

### 2.6. Measurement of ACE Activity in HUVECs

ACE activity in the treated cells was assessed using a colorimetric assay, following a previously described method [28]. This assay is based on the principle that ACE hydrolyzes the synthetic substrate hippuryl-histidine-leucine (HHL). For the assay, 200 µL of 0.01 M HHL substrate solution was mixed with 200 µL of HUVEC lysates and incubated at 37 °C for 15 min. The enzyme reaction was halted by adding 250 µL of hydrochloric acid, followed by the addition of ethyl acetate. The mixture was centrifuged, and the organic phase was transferred to a glass tube and dried at 40 °C. Acetic anhydride and p-dimethylamino-benzaldehyde were then added, and the mixture was incubated at 40 °C for 40 min. Absorbance was measured at 490 nm using a colorimetric microplate reader.

### 2.7. Determination of AT1R Protein Expression in HUVECs Using Immunocytochemistry

HUVECs were washed with phosphate-buffered saline, fixed with 4% paraformaldehyde for 10 min, and permeabilized with 0.5% Triton X-100 (Sigma, Livonia, MI, USA). The cells were then incubated in a blocking buffer to prevent non-specific binding. Primary AT1R antibody (Elabscience, Houston, TX, USA), diluted 1:50, was added onto parafilm dots, and coverslips were placed on the dots for incubation overnight at 4 °C. After incubation, the coverslips were washed and transferred onto parafilm dots containing secondary antibodies (Elabscience, Houston, TX, USA), also diluted 1:50, and incubated for 1 h in the dark. The cells were then stained with DAPI (Elabscience, Houston, TX, USA) for 10 min for nuclear staining and mounted onto glass slides using an anti-fluorescence quenching agent. The cells were observed using an Axio Vert.A1 fluorescence microscope (Carl Zeiss, Oberkochen, Germany). The images were then analyzed using ImageJ software version 8 (ImageJ Software, National Institute of Health, Bethesda, MD, USA).

### 2.8. Ex Vivo Aortic Ring Assay

Animal sacrifice and tissue collection were performed in accordance with the guidelines approved by the Universiti Kebangsaan Malaysia Animal Ethics Committee. Adult Sprague Dawley rats, aged 8 weeks and each weighing about 250 g, were euthanized by intravenous administration of a ketamine and xylazine cocktail (0.2 mL/kg). The thoracic aorta was excised, carefully cleaned of fat and connective tissue, and divided into segments of 2–3 mm segments of endothelium-intact aortic rings. These aortic rings were mounted on a wire myograph (Danish Myo Technology, Ann Arbor, MI, USA) set to optimal tension (9.8 mN). The aortic rings were kept in Krebs solution at 37 °C and continuously aerated with a mixture of 95% O_2_ and 5% CO_2_ [29]. Some rings were pretreated with 0.1–1.0 mg/mL AEPM or 0.5 µg/mL captopril for 1 h. Contraction of the aortic rings was induced using 40 mM KCl, and the results were recorded using a PowerLab Data Acquisition System (ADInstruments, Bella Vista, Australia). Cumulative contraction responses to PMA, in concentrations ranging from 10^−9^ to 10^−6^ M, were then measured. The aortic contraction responses were presented as the percentage increase in KCl-induced contraction.

### 2.9. Statistical Analysis

The data were analyzed using GraphPad PRISM 9.0. The normality of all data sets was assessed using the Shapiro–Wilk test, confirming a normal distribution. Results were presented as mean ± standard error of the mean (SEM). Differences between groups were evaluated using one-way analysis of variance (ANOVA) followed by a post hoc Tukey test. Statistical significance was set at *p* < 0.05.

## 3. Results

### 3.1. Effects of AEPM on ACE mRNA Expression and Protein Levels in PMA-Induced HUVECs

**Figure 1:**
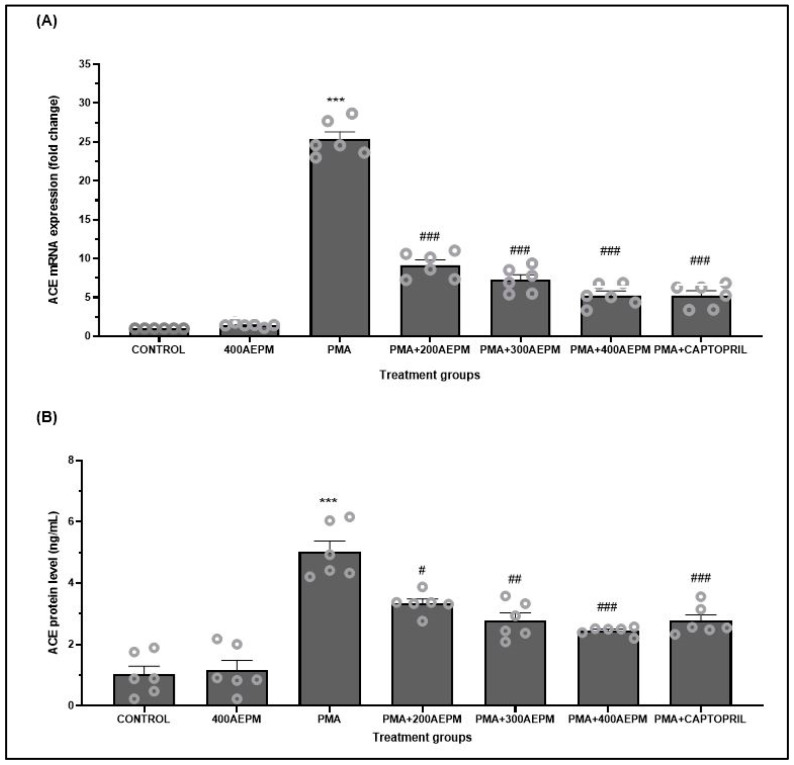
Effects of standardized aqueous extract of *P. minor* leaf (AEPM; 200, 300, and 400 µg/mL) on (**A**) angiotensin-converting enzyme (ACE) mRNA expression and (**B**) ACE protein levels in phorbol 12-myristate-13-acetate (PMA)-induced HUVECs. Data are presented as mean ± SEM, *n* = 6. *** *p* < 0.001 vs. control, ^#^ *p* < 0.05 vs. PMA, ^##^ *p* < 0.01 vs. PMA, ^###^ *p* < 0.001 vs. PMA.

Treatment with AEPM alone did not significantly affect ACE mRNA expression (Figure 1A) or protein levels (Figure 1B) compared to the control group. In contrast, HUVECs induced with PMA exhibited increased ACE mRNA expression and protein levels compared to the control group (*p* < 0.001). When PMA-induced HUVECs were treated with AEPM at doses of 200, 300, and 400 µg/mL, there was a dose-dependent reduction in ACE mRNA expression (*p* < 0.001) and ACE protein levels (*p* < 0.05, *p* < 0.01, and *p* < 0.001, respectively) compared to the PMA group. Additionally, captopril treatment also lowered ACE mRNA expression (*p* < 0.001) and protein levels (*p* < 0.001) compared to the PMA group. No significant differences in ACE mRNA expression and protein levels were found between the AEPM-treated groups and the captopril-treated group.

### 3.2. Effects of AEPM on ACE Activity in PMA-Induced HUVECs

**Figure 2:**
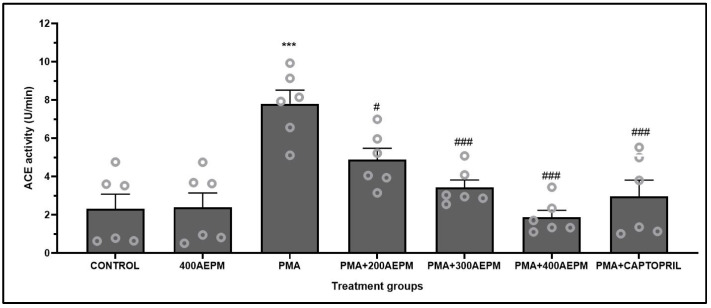
Effects of standardized aqueous extract of *P. minor* leaf (AEPM; 200, 300, and 400 µg/mL) on angiotensin-converting enzyme (ACE) activity in phorbol 12-myristate-13-acetate (PMA)-induced HUVECs. Data are presented as mean ± SEM, *n* = 6. *** *p* < 0.001 vs. control, ^#^ *p* < 0.05 vs. PMA, ^###^ *p* < 0.001 vs. PMA.

AEPM treatment alone did not result in a significant change in ACE activity compared to the control group (Figure 2). Following PMA induction, ACE activity was increased compared to the control group (*p* < 0.001). In PMA-induced HUVECs, treatment with AEPM at 200, 300, and 400 µg/mL resulted in a dose-dependent reduction in ACE activity compared to the PMA group (*p* < 0.05 and *p* < 0.001). Furthermore, captopril treatment also reduced ACE activity compared to the PMA group (*p* < 0.001). The AEPM-treated groups and the captopril group showed no significant difference in ACE activity.

### 3.3. Effects of AEPM on Angiotensin II Levels in PMA-Induced HUVECs

**Figure 3:**
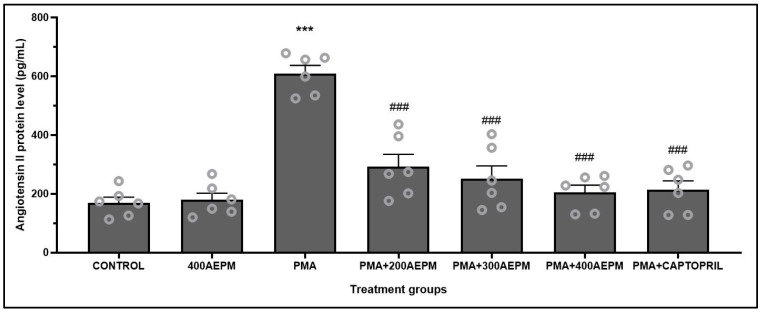
Effects of standardized aqueous extract of *P. minor* leaf (AEPM; 200, 300, and 400 µg/mL) on angiotensin II levels in phorbol 12-myristate-13-acetate (PMA)-induced HUVECs. Data were expressed as mean ± SEM, *n* = 6. *** *p* < 0.001 vs. control, ^###^ *p* < 0.001 vs. PMA.

HUVECs treated with AEPM alone showed no significant change in Ang II levels compared to the control group (Figure 3). After PMA induction, Ang II levels were markedly higher than in the control group (*p* < 0.001). Compared to the PMA group, there was a dose-dependent reduction in Ang II levels in PMA-induced HUVECs treated with 200, 300, and 400 µg/mL AEPM (*p* < 0.001). Similarly, captopril treatment also reduced Ang II levels compared to the PMA group (*p* < 0.001). The captopril group and AEPM-treated groups had comparable levels of Ang II, with no significant differences observed.

### 3.4. Effects of AEPM on AT1R mRNA Expression in PMA-Induced HUVECs

**Figure 4:**
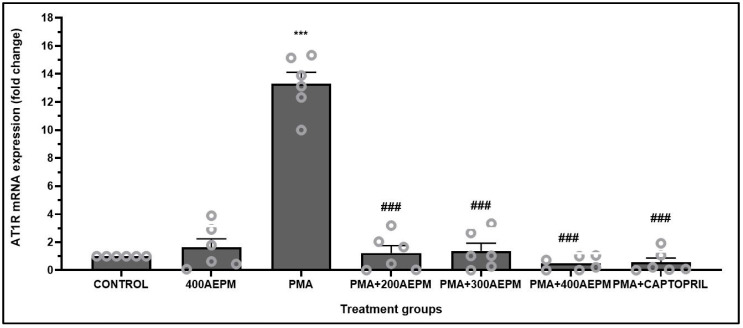
Effects of standardized aqueous extract of *P. minor* leaf (AEPM; 200, 300, and 400 µg/mL) on AT1R mRNA expression in phorbol 12-myristate-13-acetate (PMA)-induced HUVECs. Data are presented as mean ± SEM, *n* = 6. *** *p* < 0.001 vs. control, ^###^ *p* < 0.001 vs. PMA.

Treatment of HUVECs with AEPM alone did not significantly alter AT1R mRNA expression compared to the control group (Figure 4). However, AT1R mRNA expression in HUVECs was significantly upregulated following PMA induction compared to the control group (*p* < 0.001). Treatment of PMA-induced HUVECs with AEPM at concentrations of 200, 300, and 400 µg/mL successfully downregulated the AT1R mRNA expression compared to that in the PMA group (*p* < 0.001). Captopril treatment also downregulated the AT1R mRNA expression compared to the PMA group (*p* < 0.001). AT1R mRNA expression remained consistent across the AEPM-treated and captopril-treated groups, with no significant differences detected.

### 3.5. Effects of AEPM on AT1R Protein Expression in PMA-Induced HUVECs

**Figure 5:**
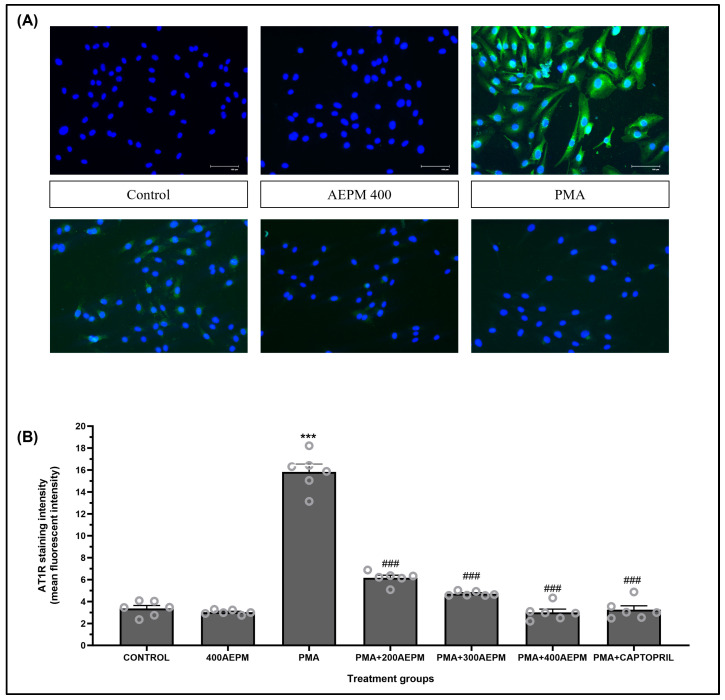
(**A**) Representative images of angiotensin II type 1 receptor (AT1R) protein expression in HUVECs. HUVECs were immunostained with antibodies against AT1R (green) and counterstained with 4′,6-diamidino-2-phenylindole (DAPI, blue) as indicated in the figure. Images were acquired at 40× magnification, scale bar: 100 µm. (**B**) Semi-quantitative analysis of AT1R staining intensity in HUVECs. Data are presented as mean ± SEM, *n* = 6. *** *p* < 0.001 vs. control, ^###^ *p* < 0.001 vs. PMA. AEPM, aqueous extract of *P. minor* leaf; PMA, phorbol 12-myristate-13-acetate.

Figure 5A shows representative images of immunocytochemical staining for AT1R protein expression in HUVECs. The cells were stained for AT1R (green fluorescence) and counterstained with DAPI (blue) to label the nuclei. As seen in Figure 5B, treatment with AEPM alone caused no significant change in AT1R expression. However, there was a significant increase in AT1R staining intensity in PMA-induced HUVECs compared to the control group (*p* < 0.001). Treatment of PMA-induced HUVECs with AEPM at concentrations of 200, 300, and 400 µg/mL resulted in a reduction in AT1R staining intensity compared to the PMA group (*p* < 0.001). Similarly, treatment of PMA-induced HUVECs with captopril also led to a significant reduction in AT1R expression compared to the PMA group (*p* < 0.001). AT1R protein expression was comparable between the AEPM-treated groups and the captopril group, with no statistically significant differences observed.

### 3.6. Effects of AEPM on Ex Vivo Aortic Contraction to PMA

**Figure 6:**
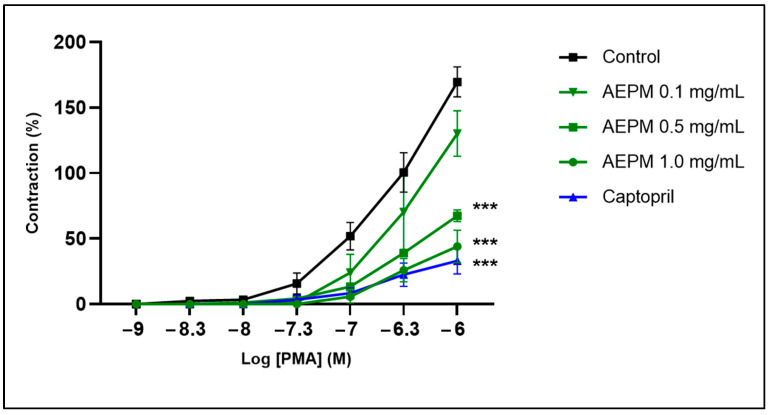
Effects of standardized aqueous extract of *P. minor* leaf (AEPM) on ex vivo aortic contraction response to phorbol 12-myristate-13-acetate (PMA). Values are presented as mean ± SEM, *n* = 6. *** *p* < 0.001 compared to the untreated control.

Figure 6 shows the contractile response of aortic rings to increasing concentrations of PMA (10^−^⁹ to 10^−^⁶ M). Aortic rings pretreated with 0.5 and 1.0 mg/mL AEPM exhibited a significantly reduced contraction response to PMA compared to the untreated control group (*p* < 0.001). Additionally, pretreatment with captopril resulted in a decreased contraction response to PMA compared to the untreated control (*p* < 0.001). The contraction response to PMA was similar between the 0.5 and 1.0 mg/mL AEPM groups and the captopril group, with no significant differences noted.

## 4. Discussion

In this study, we investigated the antihypertensive effects of AEPM via the ACE/AT1R pathway in PMA-induced HUVECs. Our results demonstrated that AEPM effectively decreased ACE mRNA expression, ACE protein levels, ACE activity, Ang II levels, and AT1R mRNA and protein expression in PMA-induced HUVECs. These in vitro findings were further supported by ex vivo experiments, where aortic rings pretreated with AEPM showed a reduced contractile response to PMA.

To determine whether AEPM has any inhibitory or stimulatory effects on the ACE/AT1R pathway in healthy endothelial cells, we treated HUVECs with AEPM alone. This treatment resulted in no significant changes in ACE mRNA expression, ACE protein levels, ACE activity, Ang II levels, or AT1R mRNA and protein expression compared to the control group. The absence of significant changes in the ACE/AT1R pathway suggests that AEPM alone does not modulate endothelial cells under basal conditions without specific triggers, potentially requiring stimuli to activate its therapeutic potential. This stability is beneficial, as the ACE/AT1R pathway plays a key role in regulating vascular tone and blood pressure, and unnecessary modulation could lead to adverse effects, such as hypotension.

In this study, treatment of HUVECs with 200 nM PMA for 24 h resulted in a significant increase in ACE mRNA expression, ACE protein levels, ACE activity, Ang II levels, and AT1R mRNA and protein expression. Previous studies have similarly shown that HUVECs exposed to 250 nM PMA for 36 h and 100 ng/mL PMA for 24 h exhibited increased ACE mRNA expression and activity [30,31]. The stimulatory effects of PMA on ACE are mediated through PKC activation, as demonstrated by experiments using PKC inhibitors [30]. PMA activates PKC by mimicking DAG, which causes PKC translocation and initiates downstream signaling. This activation triggers the mitogen-activated protein kinase (MAPK) pathways, including extracellular signal-regulated kinase (ERK) 1/2 and p38, which promote the expression of early growth response gene-1 (EGR-1) and activator protein-1 (AP-1) [32,33]. AP-1 and EGR-1 enhance ACE gene expression, leading to increased ACE protein synthesis and activity [33]. The stimulation of ACE activity by PMA led to increased Ang II production, intensifying vasoconstriction and contributing to elevated blood pressure [34]. Furthermore, a previous study has shown that elevated Ang II levels upregulate AT1R mRNA expression, creating a positive feedback loop that worsens vasoconstriction and increases blood pressure [35].

**Figure 7:**
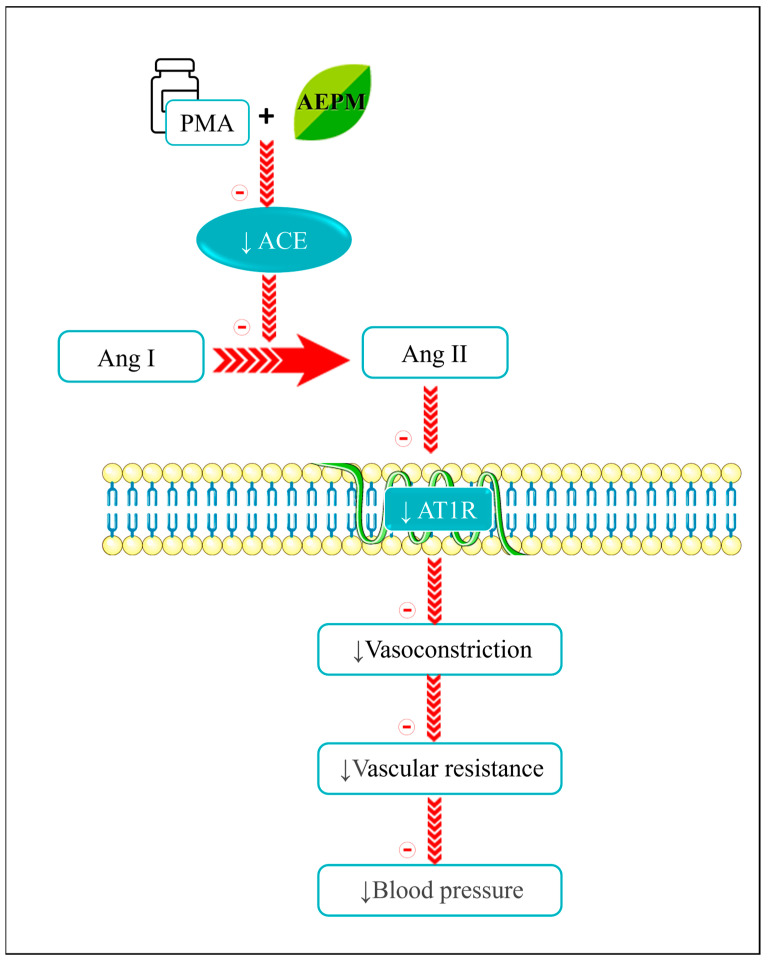
Schematic representation of AEPM’s antihypertensive mechanism through modulation of the ACE/AT1R pathway. ↓, reduced; ACE, angiotensin-converting enzyme; AEPM, aqueous extract of *P. minor*; Ang, angiotensin; AT1R, angiotensin II type 1 receptor.

In contrast, AEPM effectively suppressed ACE mRNA expression in PMA-induced HUVECs, leading to a reduction in ACE protein levels and a corresponding decrease in ACE enzymatic activity. This inhibition limited the conversion of Ang I to Ang II, resulting in lower Ang II levels. Additionally, AEPM downregulated AT1R expression, disrupting the vasoconstrictive cycle driven by the ACE/AT1R pathway. These findings are supported by ex vivo experiments, in which pretreatment with AEPM reduced the contraction response of aortic rings to PMA. Notably, the modulatory effects of AEPM on the ACE/AT1R pathway were comparable to those of the conventional ACE inhibitor, captopril. This suggests that AEPM holds significant potential as a natural ACE inhibitor for the prevention of hypertension. The antihypertensive mechanism of AEPM through modulation of the ACE/AT1R pathway is summarized in Figure 7.

In this study, treating PMA-induced HUVECs with captopril effectively inhibited ACE mRNA levels, protein expression, and enzyme activity, while also decreasing Ang II levels and AT1R expression. ACE, a metallopeptidase enzyme with a zinc ion at its active site, is inhibited by captopril due to its sulfhydryl group, which binds to the zinc ion. This binding prevents ACE from converting Ang I to Ang II, significantly reducing the enzyme’s activity [36]. In vivo studies have also shown that captopril lowers Ang II levels in spontaneously hypertensive rats [37,38]. By inhibiting ACE, the availability of Ang II to bind to AT1R is reduced, leading to decreased vasoconstriction and lower blood pressure [39]. This underscores captopril’s multifaceted role in managing hypertension by both reducing Ang II production and relieving vascular tension.

The *P. minor* leaf extract used in this study is a standardized extract containing 0.59% quercetin-3-*O*-glucuronide and 0.27% quercitrin. Previous studies have shown that quercetin-3-*O*-glucuronide contributes to vasorelaxation and blood pressure reduction in rats, while quercitrin exhibits vasorelaxant effects on rabbit aortic rings [40,41]. Additionally, other active compounds identified in *P. minor* leaf extracts include gallic acid, quercetin, and myricetin [42,43,44]. Notably, quercetin, myricetin, and gallic acid have demonstrated ACE inhibitory and antihypertensive effects [23,45]. These bioactive compounds likely contribute to the ACE inhibitory and vascular relaxation effects of AEPM.

While this study provides valuable insights into AEPM’s antihypertensive mechanisms, it is important to acknowledge its limitations. The current findings are based on in vitro HUVEC models and ex vivo aortic ring assays, which, although informative, do not fully replicate the complexity of human physiology. Future studies should include in vivo investigations using hypertensive animal models to validate these findings in a more systemic context. Additionally, clinical trials involving hypertensive patients are crucial to confirm AEPM’s efficacy, establish optimal dosing regimens, and evaluate its pharmacokinetics and long-term safety profile.

Another limitation is the study’s primary focus on the ACE/AT1R pathway. The role of the angiotensin II type 2 receptor (AT2R), which counteracts AT1R by promoting vasodilation, remains unexplored. Investigating AEPM’s impact on AT2R expression and function could provide a more comprehensive understanding of its modulation of the RAS and its potential to balance vasoconstrictive and vasodilatory forces. Additionally, exploring the effects of AEPM on other hypertensive pathways, such as endothelin-1 and nitric oxide signaling, could further elucidate its multifaceted role in vascular health.

This study employed a preventive model, where AEPM was administered concurrently with PMA, offering insights into its potential to prevent or mitigate the early stages of hypertension. However, a treatment model where AEPM is administered after endothelial activation would more closely mimic clinical scenarios. Such studies could provide a clearer picture of AEPM’s therapeutic potential for managing established hypertension, complementing its preventive benefits.

Moreover, AEPM’s potential synergistic effects with conventional antihypertensive drugs, such as ACE inhibitors or calcium channel blockers, warrant exploration. Such combinations could enhance efficacy, reduce drug dosages, and minimize side effects, providing a more holistic approach to hypertension management.

## 5. Conclusions

This study highlights *P. minor*’s ability to inhibit the ACE/ATIR pathway by decreasing ACE mRNA expression, ACE protein levels, ACE activity, Ang II levels, and AT1R expression in PMA-induced HUVECs. Therefore, *P. minor* holds potential as a natural source of ACE inhibitors for the prevention and complementary treatment of hypertension. However, further studies are essential to validate its therapeutic potential. These should include comprehensive in vivo research to confirm its efficacy in whole-organism models and clinical trials to evaluate its safety, tolerability, and long-term effectiveness in regulating blood pressure. Moreover, investigations into its bioavailability, pharmacokinetics, and possible interactions with existing antihypertensive drugs will be crucial to fully understanding its role in hypertension management.

## Figures and Tables

**Table 1:** Forward and reverse primers for qPCR.

Gene	GenBank Accession No.	Type	Sequence
Angiotensin-converting enzyme (ACE)	NM_000789	Forward Reverse	5′-atg tag atg cag ggg act cg-3′ 5′-agg gca cca cca agt cat ag-3′
Angiotensin II type 1 receptor (AT1R)	NM_032049	Forward Reverse	5′-gca caa tgc ttg tag cca aa-3′ 5′-ggg ttg aat ttt ggg act ca-3′
Glyceraldehyde-3-phosphate dehydrogenase (GAPDH)	NM_002046	Forward Reverse	5′-tcc ctg agc tga acg gga ag-3′ 5′-gga gga gtg ggt gtc gct gt-3′

## Data Availability

The original contributions presented in the study are included in the article; further inquiries can be directed to the corresponding author.

## Supplementary Materials

The following supporting information can be downloaded at https://www.mdpi.com/article/10.3390/life14111486/s1. Figure S1: High-performance liquid chromatography profile of standardized aqueous extract of *P. minor* leaves.
