# Supplementary material for: Persicaria minor (Huds.) Opiz Exhibits Antihypertensive Effects by Inhibiting the Angiotensin-Converting Enzyme/Angiotensin II Type 1 Receptor Pathway in Human Endothelial Cells
Source: Life (Basel). 2024 Nov 14;14(11):1486. doi: 10.3390/life14111486 (PMC11595449; doi:10.3390/life14111486)
Supplement: Supplementary file 1 [file life-14-01486-s001.zip › Supplementary 1.pdf]

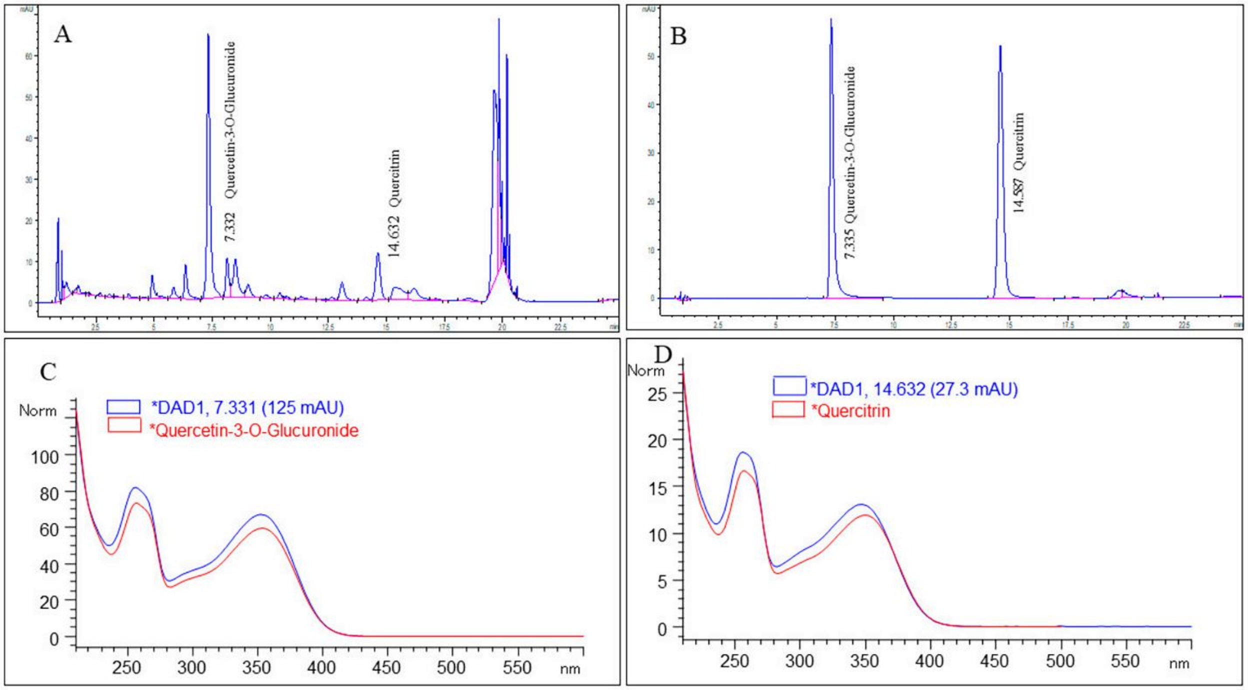

Figure S1: High-performance liquid chromatography profile of standardized aqueous extract of *P. minor* leaves. (**A**) The major peaks correspond to quercetin-3-*O*-glucuronide at retention time (R_t) 7.332 min and quercitrin at R_t 14.632 min, respectively. (**B**) Quercetin-3-*O*-glucuronide and quercitrin standards eluted at R_t 7.335 min and 14.597 min. (**C**) UV spectrum of quercetin-3-*O*-glucuronide with respect to its R_t. (**D**) UV spectrum of quercitrin with respect to its R_t.
